# Ruptured Aneurysm of Right Sinus of Valsalva in Pregnancy-A Case Report

**Published:** 2009-02

**Authors:** Leena Goel, Pooja Gautam, Suchith C

**Affiliations:** 1 Lecturer; 2 P.G.Student; 3 P.G.Student

**Keywords:** Ruptured aneurysm of right sinus of Valsalva, Pregnancy, Caesarean section, General anaesthesia

## Abstract

**Summary:**

A 26 year old multigravida at 36.6 weeks of gestation with ruptured aneurysm of right sinus of Valsalva was presented for caesarean section. Diagnosis was confirmed by transthoracic echocardiography. Here we present the anaesthetic management of this case posted for caesarean section.

## Introduction

Aneurysm of a sinus of Valsalva is a rare congenital cardiac defect, first described by Hope in 1839. Thurnam in 1840 reported the first case of rupture of the sinus of Valsalva.^1^ The clinical characteristics of aneurysm of sinus of Valsalva in cases described so far appear to be

- More common in patients of Asian Origin
- Male to female ratio is 3:1
- Uncommon in infancy and childhood
- Comprises approximately 0.1-3.5% of all congenital cardiac anomalies.

The mortality rate in patients with a sinus of Valsalva aneurysm in whom surgery is not performed is high within the first year after rupture. Cases of sudden death from sinus of Valsalva aneurysm most commonly involve rupture of the aneurysm with the acute onset of overwhelming congestive heart failure, cardiac tamponade, dysrhythmia, or coronary ischemia depending on the location of the aneurysm and thes subsequent flow disturbance. Size and location of the shunt are the major determinants of presentation and prognosis.

There are few documented cases of ruptured aneurysm of sinus of Valsalva in pregnancy.^1^,^2^ Various management options have been reported i.e. caesarean section under general anaesthesia,^2^ lumbar epidural anaesthesia during induction of labor and caesarean delivery,^3^ or normal vaginal delivery under medical supervision.^4^

## Case report

A 26 year old, 50Kg, multigravida with 8 months of amenorrhea was admitted to our hospital for elective lower segment caesarean section (LSCS) and ligation. The patient presented with complaints of breathlessness, chest pain and occasional palpitation at rest since last 7-8 months. She also complained swelling of both lower limbs which decreases after taking rest. She had normal vaginal delivery 3½ years back. There was history of one spontaneous abortion in the past. She complained of breathlessness and tiredness one year after vaginal delivery for which she consulted physician. She was informed that she has a heart disease and was diagnosed as ruptured aneurysm of right sinus of Valsalva. She was advised to undergo surgical correction of the heart ailment. She was also advised not to have pregnancy until her heart disease was corrected by surgery. But the patient could not afford the surgery and became pregnant. She was on oraldigoxin 0.25mg and frusemide 20mg per day, five times a week.

**Fig 1:**
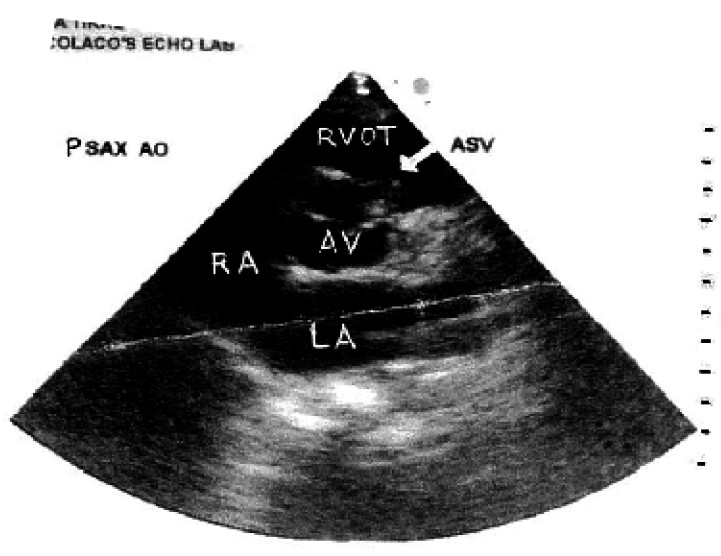
Transthoracic echocardiogram in parasternal short axis (PSAX) view shows a sinus of Valsalva aneurysm with rupture. A membranous out pouching of the right coronary cusp (RCC-arrow) protruding into the right ventricle outflow tract (RVOT) with small perforation. LA=Left atrium, RA=Right atrium, AO=Aortic root, AV= Aortic valve ASV= Aneurysm of sinus of Valsalva.

**Fig 2:**
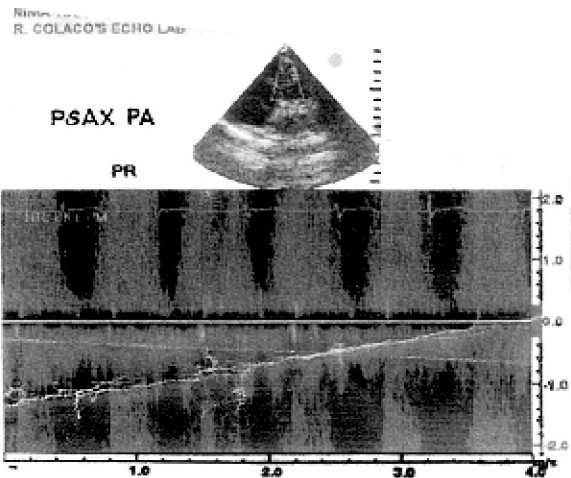
Transthoracic echocardiogram in parasternal short axis (PSAX) view shows a continuous- wave Doppler flow imaging demonstrate a continuous left to right shunt with a high-velocity systolic and diastolic flow signal. PA=Pulmonary Artery, PR=Pulmonary Regurgitation.

**Fig 3:**
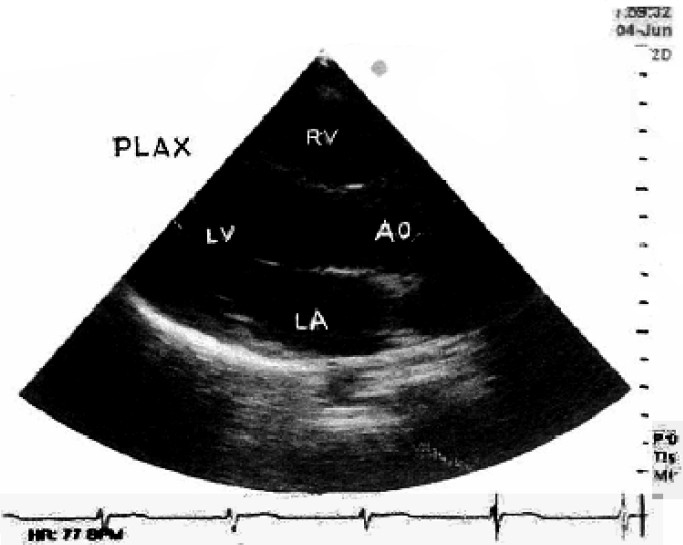
Transthoracic echocardiogram in parasternal long axis (PLAX) view shows a normal left ventricle (LV) cavity size. LA= Left atrium, AO=Aortic root, RV= Right ventricle.

On examination, her pulse rate was 96/minute, and B.P. 110/70mm of Hg. Pitting type of pedaloedema was present. Respiratory system showed bilateral equal, normal vesicular breath sounds with no rhonchi or crepitations. Onauscultation of cardiovascular system a loud continuous murmur was present over whole of the precordium. It was best heard along the lower left sternal border. A palpable thrill was present along the left sternal border. The remainder of the examination was otherwise normal for 36.6 weeks of pregnancy. Routine blood investigations were normal except Hb.(9gm%). Chest radiograph was normal. Electro-cardiogram showed sinus tachycardia. Transthoracic echocardiography (TTE) showed a membranous out pouching of the right coronary cusp (RCC) protruding into right ventricle out flow tract (RVO) with a small (3.2 mm width) perforation (Fig. 1). There was continuous wave flow with a left to right (L-R) shunt into the right (RVO) tract below the pulmonary valve (Fig. 2). The ventricular chambers were normal in size and there was good left ventricular function at rest (Fig. 3). A diagnosis of perforated aneurysm of right sinus of Valsalva was confirmed. Patient was continued with oral digoxin and lasix.

Patient was admitted for safe confinement and for elective caesarean section keeping in mind her poor cardiac status. High risk consent for anaesthesia was taken. The patient was kept fasting for six hours. The patient was shifted to operation room in propped up position breathing oxygen by apolymask. Intravenous access was obtained. Intravenous ranitidine 50mg and metoclopramide 10mg were given 30 minutes prior to induction for aspiration prophylaxis. Antibiotic prophylaxis was obtained with 2gm of intravenous amoxycillin. Monitoring of pulse rate, electrocardiography, oxygen saturation, EtCO_2,_ urine output and non-invasive blood pressure was started and continued throughout the surgery. Central venous access was obtained via the right internal jugular vein. Central venous pressure (CVP) was monitored throughout the surgery and was kept within normal limits. Rapid sequence induction was carried. Patient was preoxygenated with 100% oxygen for 5minutes. Anaesthesia was induced with thiopentone sodium (1.25%) 5mg.Kg^−1^ slowly, succinylcholine 80mg and i.v. lidocaine 1.5mg.Kg^−1^. Patient was intubated with no.7.5 oral, cuffed endotracheal tube. Lungs were ventilated with 50% O_2_ and 50% N_2_O and isoflurane (0.4%) till the birth of baby. Fifteen units of oxytocin were added to the Ringer lactate infusion after the baby was delivered. Anaesthesia was maintained with O_2_+N_2_O in the ratio 50:50, isoflurane (0.4%-0.6%), vecuronium 0.08 mg/kg and i.v. fentanyl 100mcg. EtCO_2_ was maintained around 36-40mm of Hg and CVP around 3-5 cm of water.

Intra operatively patient was hemodynamically stable with pulse rate 90-100/min. BP-130/80 mmHg and SpO_2_ 99%. Intraoperative blood loss was approximately 600ml. One crystalloid Ringer lactate was infused. Fluid replacement was guided by the CVPmonitoring. Surgery lasted for 45 minutes. Patient was reversed with neostigmine (2.5mg.) and glycopyrrolate (0.4 mg.). Lidocaine 1.5 mg. Kg^−1^ was given before extubation. Patient was shifted to the Intensive Care Unit (ICU) for observation.

## Discussion

A ruptured sinus of Valsalva aneurysm is a rare cardiac anomaly usually of a congenital nature. The sinuses of Valsalva are dilatations in the aortic wall immediately superior to the attachments of three aortic wall cusps. The sinuses are named according to their relationship with the coronary arteries: i.e. the right coronary sinus, the left coronary sinus, and the non coronary sinus.^5^ Aneurysmal dilatation of the sinuses of Valsalva occurs when the aortic media is defective, resulting in lack of fusion between aortic media and annulus fibrosus of the aortic valve.^6^ Aneurysm typically develops as a discrete flaw in the aortic media within one of the sinuses of Valsalva. Under the strain of aortic pressure, the sinus gradually weakens and dilates, causing the formation of an aneurysm. Lack of supporting tissue (e.g., ventricular septal defect) may contribute to instability and progressive distortion of the aortic sinus. Distortion and prolapsed of the sinus and aortic valve tissue can lead to progressive aortic valve insufficiency. Other cases result from an inherited connective tissue abnormality, as in Marfan syndrome or Ehlers-Danlos syndrome; from inflammatory disease, as in endocarditis, syphilitic or granulomatous aortitis, or Behcet's disease; and from mechanical disruption after stab wounds, aortic dissection, or after aortic valve replacement or ventricular septal defect repair.

The right coronary sinus is the most common site of aneurysm formation mostly ruptures into the right ventricle, producing left to right shunting.^7^ Right coronary sinus aneurysms may also rupture into the right atrium. Non coronary sinus aneurysms generally rupture into the right atrium. Left coronary sinus aneurysms are extremely rare, but they may rupture in to the pericardium, resulting in cardiac tamponade and death if not quickly recognized. Echocardiography is very accurate and reliable tool in diagnosing a sinus of Valsalva aneurysm when correlated with clinical findings.^8^ In addition Computed tomogram angiography and magnetic resonance imaging also have been reported useful in diagnosing the sinus of Valsalva.^9^‐^10^ Cine phase contrast MRI can be used for assessment of insufficiency and shunt flow.

Sinuses of Valsalva aneurysms have one of three basic pathologic patterns.^5^

- Unruptured aneurysms may cause distortion and obstruction in the right ventricular outflow tract. Dissection of the aneurysm in to the cardiac tissue may occur, causing obstruction or destruction of adjoining structures. Aneurysm may compress the interventricular septum, resulting in complete heart block with subsequent dizziness and syncope. Coronary artery compression may occur producing myocardial ischemia and chest pain. Occasionally a patient with unruptured sinus of Valsalva aneurysm presents with symptoms related to chronic aortic regurgitation.
- A slowly enlarging small perforation develops a fistulous tract in to the right ventricle and presents with a small left to right shunt. Major risk is infective endocarditis an extension of rupture with an increased shunt. Patient may remain asymptomatic for several years because of haemodynamic adjustment. However, as the degree of shunting increases symptoms related to volume overload, such as dyspnoea and exercise intolerance develops.
- An aneurysm that actually ruptures is often heralded by the sudden onset of dyspnoea and severe chest pain. Following this initial symptomatic period, the patient may become asymptomatic even without treatment as the body adjusts haemodynamically to the left to right shunting. However as the shunting and volume overload overcome the compensatory mechanisms, symptoms of congestive heart failure result.

Ruptured sinus of Valsalva connects the high pressure reservoir of the systemic circulation with the low pressure system of the pulmonary circulation resulting in a systolic-diastolic left to right shunt. There is volume overload of the left atrium, left ventricle and the aortic root that is proportional to the shunt volume. Amount of shunt volume is influenced by the diameter of the rupture and by the level of pulmonary vascular resistance. A large shunt causes a large volume of blood that is shunted from the systemic circulation, into the pulmonary circulation. The inevitable consequence of this is pulmonary congestion and rapidly developing left sided heart failure.

Differential diagnosis may be venoushum, patent ductus arteriosus, traumatic injury to the aortic root, infective endocarditis, ventricular septal defect with aortic regurgitation, truncus arteriosus with truncal valve, aortico ventricular tunnel.

## Physiological changes during pregnancy;

During normal pregnancy the total blood volume increases by 40% and plasma volume increases 45% and red blood cell volume increases by 25%, accounting for the relative anemia of pregnancy. Central Venous Pressure (CVP) and Pulmonary Capillary Wedge Pressure (PCWP) are unchanged in pregnancy.^11^ The cardiac output increases approximately by 45-50% and systemic vascular resistance decreases by 35%. This elevation in cardiac output is sustained until delivery. The rise is accomplished by an increase in both heart rate and stroke volume. In supine position aortocaval compression leads to decrease in venous return, cardiac output, and uterine blood flow after 28 weeks of pregnancy. Systolic blood pressure decreases slightly whereas significant decrease in diastolic blood pressure occurs. Associated with the expansion in blood volume is a minimal increase in left ventricular end diastolic volumes assessed by Echocardiography.^12^ Ejection fraction remains constant.

Pain and apprehension of labor further increase stroke volume and cardiac output by 45% over prelabor values.^13^ Additional stresses are imposed by uterine contractions, which cause, in effect, an autotransfusion. With each uterine contraction, central blood volume increase by 10 to 25 percent.^13^ After delivery, central blood volume also increases. Emptying of the uterus relieves obstruction of the venacava and aorta, resulting in a marked increase (up to 80% of prelabor values) in stroke volume. Therefore pregnancy is a state that places haemodynamic strain on the cardiovascular system and can be risky in woman with underlying cardiac disease.^14^ This case was typically of the congenital type and perhaps the rupture of aneurysm of sinus of Valsalva has taken placed uring vaginal delivery of earlier pregnancy. Rupture might have been precipitated by the hyperdynamic state of labor coupled with harmonically induced changes in the mechanical properties of connective tissue. Shunt diameter was not increased and there was no evidence of further rupture or there is no evidence of infective endocarditits or increase in levels of pulmonary vascular resistance. Cardiovascular system is progressively stressed during pregnancy, prelabor, and postpartum period. Increase in cardiac output will result in volume overload of left atrium, left ventricle and increase in shunt volume. Patients with limited cardiac reserve may experience cardiac failure during this time.^14^ Treatment of acute congestive cardiac failure during pregnancy aims in reducing cardiac work, bed rest, decreasing preload with diuretics, improving cardiac contractility with digitalis and other agents, reducing after load with vasodilators.^15^

In this case an elective caesarean section under general anaesthesia was planned for her since the patient was already haemodynamically compromised. The haemodynamic changes occurring during labor would have exacerbated the left to right shunt and perhaps extended the aneurismal tear. In vaginal delivery cardiac output increases 80% of the prelabor values as compared to 50% in caesarean section.^16^ Although epidural anaesthesia would have decreased the systemic vascular resistance (SVR) secondary to sympathetic nervous system blockade and would have decreased the cardiac output, nevertheless decrease in S.V.R is not predictable and not easy to control there-fore should probably not to be selected over general anaesthesia. Epidural anaesthesia is associated with an increase in only 40%in cardiac output and general anaesthesia, with an augmentation of only 25%.^17^

Our goals of management in this case were:

- Maintain haemodynamic stability and cardiac output.
- Prevent bradycardia.
- Prevent pulmonary congestion
- Prevent increase in S.V.R.

Anaesthesia was induced in supine position with 10°-15° left lateral tilt. To attenuate epressure response lidocaine 1.5mg.kg^−1^ was given prior to laryngoscopy. Although succinylcholine can abruptly increase the parasympathetic nervous system activity and could theoretically have additive effects with digitalis, we preferred succinylcholine because of our clinical experience. Anaesthesia was maintained with positive pressure ventilation as it decreases the preload thereby improving the cardiac function. Vecuronium was preferred due to better cardiac stability. Low doses of opioids were given for analgesia. The patient was monitored in ICU post operatively.

Normally there is a large increase in tota lblood volume and a significant increase in cardiac output in pregnancy which places haemodynamic strain on the cardiovascular system. This excessive burden further increases in a patient with left to right shunt resulting from ruptured aneurysm of right sinus of Valsalva. Caesarean section under general anaesthesia appears to be an optimal management of delivery in pregnancy of such patients. The definitive surgical repair of cardiac defect can be undertaken subsequently depending upon the condition of the patient.
